# Assessing anxiety in high-altitude Tibetan communities: validation of a Tibetan version GAD-7

**DOI:** 10.3389/fpsyg.2026.1807621

**Published:** 2026-06-24

**Authors:** Xuewei Fu, Xi Wang, Jiafeng Li, Gongrongxiu Cui, Dhondup Dolma, Dawa Pendo, Raphael Shi Tang, Jiajun Xu, Yunzhu Chen, Lijun Jiang

**Affiliations:** 1 Mental Health Center, West China Hospital, Sichuan University, Chengdu, China; 2 National Center for Mental Disorders, West China Hospital, Sichuan University, Chengdu, China; 3 Department of Cardiology, West China Hospital, Sichuan University, Chengdu, China; 4 Glasgow College, University of Electronic Science and Technology of China, Chengdu, China; 5 Southwest Minzu University, Chengdu, China; 6 Department of Psychiatry, The Chinese University of Hong Kong, Hong Kong, Hong Kong SAR, China

**Keywords:** cultural adaptation, GAD-7, high-altitude populations, mental health assessment, Tibetan version

## Abstract

**Objective:**

To translate the Generalized Anxiety Disorder-7 (GAD-7) scale into Tibetan and evaluate its psychometric properties for assessing anxiety symptoms of the Tibetan communities living in high altitudes.

**Methodology:**

Following a standardized translation and cross-cultural adaptation procedure, the Tibetan GAD-7 was administered to 583 adults recruited via convenience sampling in Tibetan areas. Its reliability was assessed using Cronbach’s alpha and item-total correlations. Construct validity was examined using Exploratory Factor Analysis (EFA, *n* = 290) and Confirmatory Factor Analysis (CFA, *n* = 293) by randomly splitting the sample into two halves. Group differences in GAD-7 scores across sociodemographic variables were analyzed using t-tests and Analysis of Variance (ANOVA).

**Results:**

The Tibetan GAD-7 demonstrated acceptable internal consistency (Cronbach’s *α* = 0.70). EFA suggested a two-factor structure, which was further supported by CFA, showing a significantly better model fit (CFI = 0.90, TLI = 0.82, RMSEA = 0.09) than a one-factor model (CFI = 0.71, TLI = 0.57, RMSEA = 0.14). Significant group differences were also found, with females, urban residents, and individuals with lower household income reporting higher anxiety scores.

**Conclusion:**

The Tibetan version of the GAD-7 shows acceptable reliability and validity as a screening tool for generalized anxiety symptoms in high-altitude Tibetan populations. Its use can facilitate early identification of anxiety in this underserved, culturally distinct community with limited mental health resources.

## 1 Introduction

Generalized anxiety disorder (GAD) is a chronic and disabling mental health condition characterized by excessive, uncontrollable worry and persistent psychological and somatic tension, which is associated with increased health care utilization and reduced quality of life and functioning (Caldichoury et al., 2025; Szuhany and Simon, 2022; Xue et al., 2025). Not only quality of life and function, but the symptoms also increase the risk of comorbid psychiatric and physical disorders (Allgulander and Baldwin, 2013). Despite growing recognition of anxiety disorders as a global health concern, disparities remain in detection and treatment across different cultural and linguistic groups (Li and Xu, 2024).

The Generalized Anxiety Disorder-7 (GAD-7), as a reliable scale, is a widely used self-report screening tool for GAD worldwide (Camargo et al., 2023; Zinchuk et al., 2021). Since its development, it has demonstrated strong psychometric properties, ease of administration, and utility in both clinical and community settings (Johnson et al., 2019). However, most validation studies of the GAD-7 have been conducted in Western or urban populations. Evidence on its applicability in culturally distinct, rural, and minority populations is still limited, which raises concerns about whether the measure adequately captures culturally specific expressions of anxiety.

The plateau ethnic minority population in China represents a unique cultural and linguistic group with specific sociocultural characteristics and health beliefs (Shen et al., 2011; Wang et al., 2022). Language, worldview, and cultural norms may influence how individuals perceive, express, and report symptoms of anxiety (Pashak et al., 2020; Stade et al., 2023; Zisberg, 2017). Furthermore, residents in the plateau region often face geographic and structural barriers to accessing mental health services, making brief, valid, and culturally adapted screening tools particularly important (Wang et al., 2024). Thus, without appropriate validation, the risk of underdiagnosis or misdiagnosis of anxiety symptoms in this population remains high.

In order to mitigate this gap, the present study aimed to translate and culturally adapt the GAD-7 into Tibetan and to evaluate its psychometric properties, including reliability and validity, in a large sample of plateau adults. In addition, we explored the performance of the Tibetan Language GAD-7 across different sociodemographic subgroups to assess its measurement invariance and practical utility in diverse segments of the population. Establishing the reliability and validity of the Tibetan version of the GAD-7 will provide a culturally appropriate and efficient screening instrument, supporting early identification of anxiety symptoms and informing clinical practice and public health strategies in the region.

## 2 Method

### 2.1 Measure

The Generalized Anxiety Disorder Scale-7 (GAD-7) is a widely used self-report instrument developed by Spitzer et al. (2006) to assess the severity of generalized anxiety symptoms over the past two weeks. The scale consists of seven items reflecting the core symptoms of generalized anxiety disorder as defined by the DSM-IV, including excessive worry, restlessness, irritability, and difficulty controlling anxiety. Each item is rated on a 4-point Likert scale ranging from 0 (“not at all”) to 3 (“nearly every day”), yielding a total score between 0 and 21, with higher scores indicating greater symptom severity. Conventionally, cutoff points of 5, 10, and 15 are used to represent mild, moderate, and severe anxiety, respectively. In the present study, the Tibetan version of the GAD-7 used a 1–4 Likert scale, with higher scores indicating greater anxiety symptom severity.

The original and translated versions of GAD-7 can be freely downloaded from the official PHQ website^1^. According to the website statement, no permission is required to reproduce, translate, display, or distribute the measure.

### 2.2 Translation and retranslation

In the study, the internationally recognized procedure was adopted for the cross-cultural adaptation of psychological scales. First, two researchers proficient in both Chinese and Tibetan, with backgrounds in psychology, conducted forward translation and produced the initial Tibetan draft. After an independent translator performed a back translation into Chinese, an expert panel including specialists in psychology, linguistics, and clinical medicine, compared the original and back-translated versions to examine semantic, conceptual, and cultural equivalence. After revisions, a pilot version was compiled and then pretested among a small group of Tibetan volunteers. Their feedback on comprehension was collected and incorporated into further revisions, resulting in the finalized Tibetan version of the GAD-7 scale. During translation, the response format was adapted from the original 0–3 scale to a 1–4 Likert scale to facilitate comprehension among Tibetan respondents, while preserving the original semantic anchors. The modification of the response format from the original 0–3 scale to a 1–4 scale warrants careful consideration. The modification was to improve comprehensibility among a high-altitude ethnic minority population, in which the pilot study indicated that the use of “0” as a response option was occasionally misunderstood or conflated with missing responses.

### 2.3 Data collection

A convenience sampling method was used to recruit participants in public settings in Tibetan areas, such as squares, shopping malls, and residential communities. The purpose, procedure, and confidentiality principle of the study were all explained by trained researchers, and written informed consent was obtained before completing each questionnaire. This survey was conducted on paper and provided with on-site guidance and clarification as needed. The average completion time of the questionnaires was approximately 5–10 min. They were completed anonymously, entered by professional staff, and stored in encrypted form for research purposes only, ensuring participants’ privacy and voluntary participation. This study was approved by the Ethics Committee of West China Hospital, Sichuan University (Approval No. 2026–11). All participants were voluntary and informed about the purpose of the study and provided written informed consent prior to participation, and anonymity and confidentiality were ensured throughout the study.

### 2.4 Statistical analysis

All statistical analyses were conducted using IBM SPSS Statistics 26.0 and Mplus 8.3. The significance level was set at *p* < 0.05 (two-tailed). Descriptive statistics were computed to examine the distributional characteristics of the Tibetan version of the GAD-7. Item means, standard deviations, skewness, and kurtosis were inspected to evaluate central tendency, variability, and normality, ensuring the suitability of the data for subsequent analyses.

### 2.5 Reliability analysis

Internal consistency reliability was assessed using Cronbach’s alpha coefficient based on the total scale score. Item analysis was conducted by examining corrected item-total correlations and Cronbach’s alpha if item deleted.

### 2.6 Construct validity

To examine the construct validity of the GAD-7, the total sample was randomly divided into two approximately equal subsamples. Random numbers between 0 and 1 were generated in SPSS, and participants were assigned accordingly, resulting in one subsample of 290 participants and a second subsample of 293 participants. Exploratory factor analysis (EFA) was conducted on the first subsample (*n* = 290) to identify the underlying factor structure. The EFA was performed using principal axis factoring with oblique (Promax) rotation, allowing factors to correlate. The number of factors to retain was determined based on eigenvalues greater than 1 and inspection of the scree plot. Sampling adequacy was assessed with the Kaiser–Meyer–Olkin (KMO) measure and Bartlett’s test of sphericity. Based on the EFA results, confirmatory factor analysis (CFA) was performed on the second subsample (*n* = 293) using Mplus. Given the ordinal nature of the GAD-7 items, the CFA models were estimated using the robust weighted least squares estimator (WLSMV). Model fit was evaluated using the chi-square statistic (χ^2^), Comparative Fit Index (CFI), Tucker-Lewis Index (TLI), Root Mean Square Error of Approximation (RMSEA), and Standardized Root Mean Square Residual (SRMR).

To examine measurement invariance of the scale across gender, we conducted a multiple-group CFA comparing male and female participants. Individuals identified as other were excluded due to the small sample size, which may lead to unstable parameter estimates in multiple-group CFA. The analysis proceeded in three steps: first, a configural model was tested to assess whether the factor structure was equivalent across male and female participants without constraining factor loadings or intercepts. Second, metric invariance was evaluated by constraining factor loadings to be equal across groups. Third, scalar invariance was tested by additionally constraining item intercepts to be equal. Model fit was assessed using χ^2^, RMSEA, CFI, TLI, and SRMR. Comparisons between models were conducted to evaluate whether adding constraints significantly worsened model fit.

### 2.7 Group differences

Group differences in GAD-7 total scores were analyzed using independent-sample t-tests for dichotomous variables (e.g., gender, religious affiliation) and one-way analyses of variance (ANOVA) for variables with more than two categories (e.g., age, education, occupation, marital status, residence, and household income). Bonferroni-corrected *post hoc* tests were applied following significant ANOVA results to identify specific between-group differences.

## 3 Results

### 3.1 Sample characteristics

A total of 583 participants were recruited and completed the Tibetan version of GAD-7. Gender distribution was relatively balanced, with 46.8% identifying as male, 44.4% as female, and 8.7% as other. Nearly half of the sample (47.5%) were aged 18–24 years, followed by 25–34 years (26.8%) and 35–44 years (16.6%), with smaller proportions in older age groups. Overall, participants came from diverse educational and occupational backgrounds, varying marital statuses, and a wide range of residential and economic conditions, ensuring a heterogeneous and representative sample. Detailed demographic characteristics are presented in Table 1.

**Table 1:** Sample characteristics of the participants (*N* = 583).

Variable	*N* (%)
Gender	
Male	273 (46.8)
Female	259 (44.4)
Other	51 (8.7)
Age (years)	
18–24	277 (47.5)
25–34	156 (26.8)
35–44	97 (16.6)
45–54	32 (5.5)
≥55	21 (3.6)
Education level	
Primary school or below	216 (37.0)
Secondary school	131 (22.5)
Diploma	147 (25.2)
Bachelor’s degree	64 (11.0)
Master’s or above	25 (4.3)
Occupation	
Student	251 (43.1)
Employee	107 (18.4)
Self-employed	109 (18.7)
Unemployed	56 (9.6)
Retired	23 (3.9)
Other	37 (6.3)
Marital status	
Single	296 (50.8)
Married	157 (26.9)
Divorced	103 (17.7)
Widowed	27 (4.6)
Type of residence	
Urban	209 (35.8)
Suburban	211 (36.2)
Rural	163 (28.0)
Household income (monthly, RMB)	
< 3,000	261 (44.8)
3,000–6,000	184 (31.6)
6,001–10,000	104 (17.8)
> 10,000	34 (5.8)

### 3.2 Descriptive statistics

Descriptive statistics were calculated for the Tibetan version of the GAD-7. The total score ranged from 7 to 28, with a mean of 14.58 (SD = 4.53). Item means ranged from 1.61 to 2.46, and standard deviations ranged from 0.99 to 1.23, indicating moderate variability across items. Skewness values (0.01 to 1.51) and kurtosis values (−1.58 to 0.89) fell within acceptable thresholds, suggesting that the score distributions approximated normality and were suitable for subsequent analyses.

### 3.3 Internal consistency

The internal consistency of the Tibetan version of the GAD-7 was acceptable, with a Cronbach’s *α* of 0.70. Corrected item–total correlations were all positive and ranged from 0.36 to 0.45, indicating adequate item discrimination. Removal of any single item did not result in a meaningful improvement in internal consistency, supporting the coherence of the scale (Table 2).

**Table 2:** Item-level descriptive statistics and item analysis of the Tibetan GAD-7.

Item	Mean	SD	Corrected item-total correlation	Cronbach’s α if item deleted
1	1.61	1.01	0.39	0.67
2	2.13	0.99	0.40	0.67
3	2.08	1.08	0.45	0.65
4	2.46	1.23	0.36	0.68
5	2.04	1.07	0.43	0.66
6	2.15	1.05	0.42	0.67
7	2.10	1.14	0.42	0.67

Items were rated on a 4-point Likert scale ranging from 1 to 4.

Corrected item–total correlations refer to correlations between each item and the total score excluding that item.

Cronbach’s α if item deleted indicates the internal consistency of the scale when the corresponding item is removed.

### 3.4 Construct validity

To examine the underlying structure of the Tibetan GAD-7, an EFA was conducted. Sampling adequacy was confirmed by a KMO value of 0.71, and Bartlett test of sphericity was significant, χ^2^ (21) = 438.87, *p* < 0.001. Based on eigenvalues greater than one and theoretical considerations, a two-factor solution was retained. The first factor accounted for 30.44% of the common variance, and the second factor accounted for 10.31%, explaining a total of 40.75% of the extracted variance. The pattern matrix demonstrated a clear and interpretable structure, with all items loading strongly on their respective factors (standardized loadings ≥ 0.42) and no substantial cross-loadings observed. Items 2, 4, and 6 loaded more strongly on the second factor, while the remaining (items 1, 3, 5, 7) loaded primarily on the first factor, suggesting a potential two-factor structure that may reflect different dimensions of anxiety symptoms.

(CFA) was conducted on the second subsample (*n* = 293) using Mplus. Two models were specified and tested: a one-factor model in which all seven items loaded onto one latent factor, and a two-factor model in which items 2, 4, 6 loaded onto the first factor, and items 1, 3, 5, 7 loaded onto the second factor.

**Figure 1:**
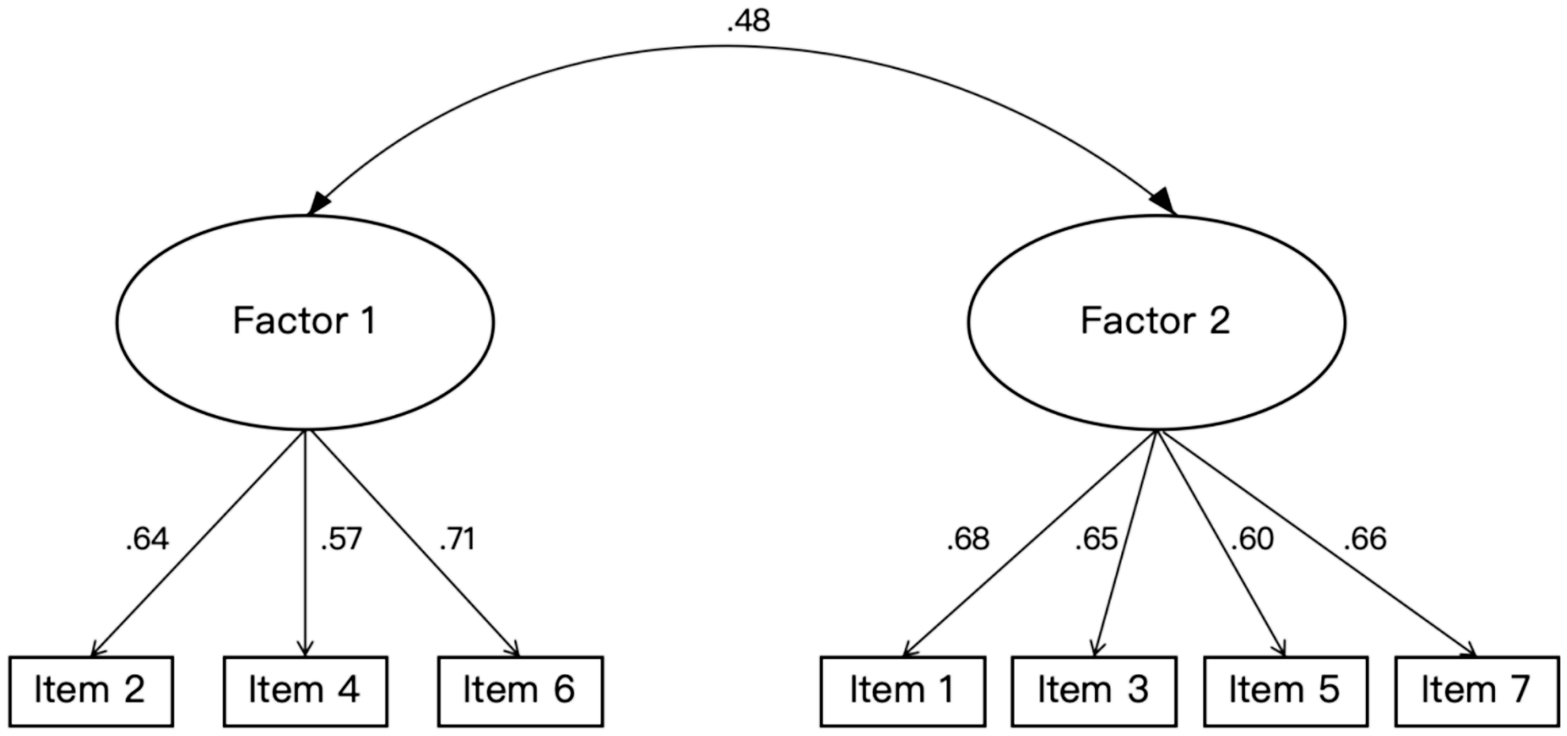
Standardized solution of the two-factor confirmatory factor analysis model for the Tibetan version of the GAD-7. Rectangles represent observed items and ellipses represent latent factors. Values on the single-headed arrows indicate standardized factor loadings, and the curved double-headed arrow represents the correlation between the two factors.

For the one-factor model, the fit indices indicated suboptimal fit: χ^2^ (14) = 108.18, *p* < 0.001, RMSEA = 0.15 (90% CI [0.13, 0.18]), CFI = 0.78, TLI = 0.67, and SRMR = 0.09. Standardized factor loadings ranged from 0.46 to 0.60, with items 4 and 6 exhibiting relatively lower loadings compared to the other items. The two-factor model demonstrated improved fit: χ^2^ (13) = 35.94, *p* < 0.001, RMSEA = 0.08 (90% CI [0.05, 0.11]), CFI = 0.96, TLI = 0.91, and SRMR = 0.05. In this model, items 2, 4, and 6 loaded on Factor 1 (loadings = 0.57–0.71), whereas items 1, 3, 5, and 7 loaded on Factor 2 (loadings = 0.60–0.68). The correlation between the two factors was moderate (r = 0.48), indicating substantial shared variance while still reflecting distinguishable item clustering. Compared with the one-factor model, the two-factor model exhibited lower χ^2^ and RMSEA, higher CFI and TLI, smaller SRMR, and the pattern of standardized loadings revealed clearer item clustering (Figure 1).

Measurement invariance across gender was examined using multiple-group confirmatory factor analysis. The configural model demonstrated marginally acceptable fit (χ^2^ = 104.16, df = 26, *p* < 0.001; RMSEA = 0.106; CFI = 0.889; TLI = 0.821; SRMR = 0.050). The metric model did not show a significant decrease in fit compared to the configural model (Δχ^2^ = 8.24, Δdf = 5, *p* = 0.144; ΔCFI < 0.01) and demonstrated comparable fit (CFI = 0.885; RMSEA = 0.099). Similarly, the scalar model was supported, with no significant deterioration relative to the metric model (Δχ^2^ = 1.23, Δdf = 5, *p* = 0.942; ΔCFI < 0.01) and acceptable fit (CFI = 0.890; RMSEA = 0.090). These findings support configural, metric, and scalar invariance across male and female participants (Table 3).

**Table 3:** CFA model fit indices for the Tibetan GAD-7.

Model	χ^2^	df	χ^2^/df	CFI	TLI	RMSEA (90% CI)	SRMR
One-factor model	108.18	14	7.73	0.78	0.67	0.15 [0.13, 0.18]	0.09
Two-factor model	35.95	13	2.77	0.96	0.91	0.08 [0.05, 0.11]	0.05

χ^2^ = chi-square; df = degrees of freedom; CFI = comparative fit index; TLI = Tucker–Lewis index; RMSEA = root mean square error of approximation; SRMR = standardized root mean square residual.

### 3.5 Group differences

GAD-7 total scores differed significantly across gender, place of residence, and household income. Females reported higher scores than males (t = −2.31, *p* = 0.021). Participants living in urban areas had higher scores compared with those in suburban (mean difference = 1.34, *p* = 0.007) or rural areas (mean difference = 1.25, *p* = 0.023). Regarding household income, participants earning less than 3,000 yuan per month had higher scores than those earning 6,001–10,000 yuan (mean difference = 1.72, *p* = 0.006) and more than 10,000 yuan (mean difference = 3.12, *p* < 0.001). No significant differences in GAD-7 scores were observed across age groups (*F* (4, 578) = 1.63, *p* = 0.165), educational levels (*F* (4, 578) = 1.16, *p* = 0.328), marital status (*F* (3, 579) = 1.85, *p* = 0.137), occupational status (*F* (5, 577) = 1.63, *p* = 0.157), or religious affiliation (t = −0.29, *p* = 0.386).

## 4 Discussion

The present study has developed the Tibetan version of GAD-7 and examined its psychometric properties and latent structure among ethnic minority populations residing in high-altitude plateau regions of China.

Internal consistency of the Tibetan GAD-7, assessed in the full sample, reached the conventional threshold for group-level research, with a Cronbach’s *α* of 0.70. All item-total correlations were positive and fell within the moderate range, indicating that the seven items function coherently as a set. Although the internal consistency observed in this study is somewhat lower than that reported in some prior validations conducted in general or clinical populations, such as an *α* = 0.89 in the German general population(Löwe et al., 2008) or α = 0.92 in primary care settings in Latvia(Vrublevska et al., 2022), it is comparable to findings from studies (e.g., α = 0.77 in university students in Ethiopia or α = 0.76 in a study of Arabic-speaking Saudi in primary care clinics) conducted in culturally and linguistically diverse settings(AlHadi et al., 2017; Manzar et al., 2021). Several factors may help explain the relatively modest internal consistency. First, Cronbach’s α is sensitive to the number of items, and shorter scales such as the GAD-7 are more likely to yield lower coefficients. Second, the two-factor structure identified in the present study may have contributed to this finding, as Cronbach’s α assumes unidimensionality and may underestimate reliability when multiple related dimensions are present. In addition, cultural and contextual factors may also play a role. In high-altitude Tibetan communities, anxiety is often expressed with greater somatic salience — for example, through sleep disturbances, fatigue, or cardiovascular symptoms — rather than predominantly affective or cognitive complaints. Cross-cultural studies have documented that non-Western populations often report more somatic symptoms when experiencing anxiety, a pattern that can weaken inter-item correlations on scales developed in Western contexts (Hofmann and Hinton, 2014). In high-altitude Tibetan students, environmental stressors such as hypoxia and sleep disruption have been associated with elevated somatic symptom reporting alongside anxiety, suggesting that both physiological and cultural factors may increase heterogeneity in item responses and reduce internal consistency estimates like Cronbach’s *α* (Shi et al., 2025). Taken together, these findings suggest that the observed level of internal consistency does not necessarily indicate inadequate reliability, while suggesting that further refinement or contextual calibration may enhance its performance.

Using a split-sample design, exploratory and confirmatory factor analyses indicated that the Tibetan GAD-7 is better represented by a two-factor structure, showing better model fit than one-factor model, with items clustering into two distinguishable but moderately correlated groups. This pattern suggests that, while the scale functions effectively as a global measure of generalized anxiety (Hinz et al., 2017; Riglea et al., 2025), its items reflect subtle substructure rather than a strictly unidimensional latent construct. Similar findings have been reported in several clinical or context-specific samples, where alternative specifications—such as two-factor, bifactor, or modified one-factor models with correlated residuals—provided improved fit relative to strictly unidimensional models (Brattmyr et al., 2022; Dhira et al., 2021; Johnson et al., 2019; Kigozi, 2021).

In the Tibetan version of the GAD-7, translation and linguistic factors may have introduced subtle differences in how individual items are interpreted. Items 2, 4, and 6 include expressions corresponding to notions such as “cannot,” “difficulty,” or “ease,” whereas items 1, 3, 5, and 7 are more likely to be rendered using terms related to internal experiences, such as “unease,” “worry,” or “fear.” These wording differences may lead respondents to process the two groups of items in systematically distinct ways, contributing to the emergence of a two-factor structure. This pattern may be particularly meaningful in high-altitude ethnic minority populations, where anxiety symptoms manifest through intertwined cognitive, emotional, and somatic channels shaped by environmental stressors, including chronic physical strain, hypoxia-related effects, and limited access to mental health resources (Adams and Peel, 2025; Ge, 2025). Within the Tibetan cultural context, conceptualizations of mind and mental illness often rely on traditional frameworks such as rlung (“wind”), integrating physiological, emotional, and spiritual dimensions rather than isolating discrete constructs such as “anxiety” (Deane, 2019). Psychological distress is understood holistically, with emotional experiences, bodily states, and spiritual balance considered interdependent. Influenced by Buddhist perspectives, suffering may be viewed as an inherent aspect of human existence, and emotional experiences may be interpreted as causal or contributing factors rather than as discrete pathological symptoms (Desai and Chaturvedi, 2017; Kaiser and Jo Weaver, 2019; Lewis, 2021; Gentsch and Kuehn, 2022; Stein et al., 2024). These culturally mediated frameworks, together with linguistic translation effects and structural limitations of Western-derived assessment tools, may help explain why the GAD-7 items grouped into two factors, reflecting patterned ways of experiencing and reporting distress rather than distinct latent anxiety dimensions in the Western sense (Kohrt et al., 2014; Kirmayer, 1989).

From a methodological perspective, in brief self-report measures such as the GAD-7, where each domain is represented by only a small number of items, even modest residual associations among items with similar experiential focus may contribute to the emergence of separable factors in model estimation. In the present study, shared features within each item cluster may contribute to covariance beyond the general anxiety factor. Furthermore, the moderate correlation between the two factors suggests that these clusters are not independent dimensions, but rather reflect related facets of a broader anxiety construct. Thus, the observed two-factor structure may, in part, capture domain-specific variance embedded within a largely unidimensional construct, rather than indicating fully distinct psychological dimensions.

Beyond factor structure and reliability, the study also identified demographic differences in the high-altitude plateau population using Tibetan GAD-7. Females reported higher anxiety levels than males, consistent with extensive evidence documenting higher prevalence and severity of anxiety symptoms among women across cultures (Farhane-Medina et al., 2022; Kaluve et al., 2022). Participants residing in urban areas reported higher anxiety scores than those living in suburban or rural areas, which may reflect greater psychosocial stress, faster-paced living conditions, and heightened economic or social pressures in urban settings, even within plateau regions. In addition, lower household income was associated with higher anxiety severity, highlighting the role of financial insecurity as a chronic stressor. By comparison, no significant differences were observed across age groups, educational levels, marital status, occupational status, or religious affiliation, suggesting that the GAD-7 performs consistently across these subgroups and supports its broad applicability in this population.

During translation, the response format was adapted from the original 0–3 scale to a 1–4 Likert scale to facilitate comprehension among Tibetan respondents, while preserving the original semantic anchors. From a psychometric perspective, this change represents a linear transformation and is therefore unlikely to substantially affect the underlying factor structure or internal consistency of the scale. However, it may have implications for score interpretation and comparability with existing studies using the original scoring system. In particular, established cutoff scores for the GAD-7 (e.g., 0–4 represents minimal, 5–9 mild, 10–14 moderate, and 15–21 severe) cannot be directly applied. For cross-study comparisons, score standardization or linear rescaling is recommended.

Several limitations of the present study should be acknowledged. First, participants were recruited through convenience sampling. Although the sample included variation in age, education, and occupational backgrounds, this approach limits representativeness and the generalizability of the findings. Replication in larger plateau populations and other Tibetan-inhabited areas is therefore necessary to confirm the applicability of the results. Second, the cross-sectional design precluded assessment of test–retest reliability, preventing evaluation of the temporal stability of the scale. Third, the study did not compare the Tibetan GAD -7 with other established psychological measures, limiting assessment of external validation. Additional evidence is needed to determine how well the Tibetan GAD-7 aligns with related constructs. Fourth, the study did not include structured clinical diagnostic assessments or ROC analysis, which limited the ability to evaluate criterion validity and diagnostic utility. Future studies incorporating structured clinical interviews will be essential to assess diagnostic accuracy, establish appropriate cutoff points, and enhance the practical utility of the Tibetan GAD-7 in screening contexts. Finally, although the present study identified a two-factor structure, the analyses were based on a single dataset and modeling approach, and alternative specifications were not formally tested. As such, the extent to which the observed factor structure reflects stable underlying dimensions, as opposed to context-specific or methodological influences, remains to be further clarified. Overall, these limitations suggest that, while the present findings provide preliminary support for the reliability and structural validity of the Tibetan GAD-7, the interpretation of its factor structure and broader applicability should be made with consideration of both methodological constraints and contextual influences.

In conclusion, the Tibetan GAD-7 demonstrated acceptable internal consistency and an overall satisfactory model fit, providing initial evidence of its construct validity with the ethnic minority population in high-altitude regions of China. Given the limited access to mental health services and constrained referral pathways in these areas, the availability of a brief, easy-to-administer, and culturally adapted tool is particularly important. Within this context, the present findings offer a preliminary empirical foundation for the use of the Tibetan GAD-7 in this population. Future research could build upon these results by incorporating external validation, including comparisons with related psychological measures and clinical diagnostic assessments, in order to further establish its convergent and criterion validity. Such efforts would contribute to the development of a more robust and contextually appropriate screening tool for assessing anxiety symptoms in high-altitude populations.

## Data Availability

The original contributions presented in the study are included in the article, further inquiries can be directed to the corresponding authors.

## Footnotes

1
https://www.phqscreeners.com/
